# Bibliographical Notices

**Published:** 1847-07

**Authors:** 


### BIBLIOGRAPHICAL NOTICES.

*Lehrbuch der Azneimittellehre.* Von Dr. C. G. Mitscherlich, Privatdocenten an der Königl. Friedrich-Wilhelms-Universität und praktischem Arzte zu Berlin. 8vo. 2 *Band.* Berlin, 1838, 1846.

*Manual of Materia Medica.* By C. G. Mitscherlich, Privatim docens to the Royal Frederick-William's University, and practical Physician at Berlin. Two volumes. Berlin. 1838.

*Annuaire de Thérapeutique, de Matière Médicale, de Pharmacie et de Toxicologie pour* 1847, *contenant le Resumé des Travaux Thérapeutiques et Toxicologiques publiés en* 1846, *et les Formules des Médicaments nouveaux; suivi d'un Mémoire sur les principaux contrepoisons, et sur la Thérapeutique des empoisonnements et de diverses notices scientifiques.* Par le Dr. A. Bouchardat, Chevalier de la Legion d'Honneur, Agrégé de la Faculté de Médecine de Paris, Pharmacien en chef de l'Hôtel Dieu, &c. 18mo. pp. 302. Paris, 1847.

Our German brethren have a singular mode of bringing out many of their treatises on science, and on medical science more especially. Mitscherlich's work on Materia Medica appeared in its first portion as long ago as the year 1838, and its last has not been long issued. There can, therefore, be but little unity in such productions; and it must often happen, that the purchaser of the first parts must be disappointed in receiving the remainder, and, when he does obtain them, they cannot be in entire keeping with the precursors; as, generally, in the lapse of years, material changes must have occurred in the views of the author, and in the progress of science. In most works of a cyclopædiac character, time—and a long time—must necessarily elapse before they can be concluded, and the same objection holds as to the want of uniformity in the earlier and later articles; but as these are not necessarily—nor perhaps often—by the same author, the objection is not so valid. In the case of Dr. Copland's valuable dictionary, written wholly by himself, great and deplorable delay has occurred, and—as we have before said—there is no knowing when it will terminate; and if the author's life should unfortunately end, and science be thus deprived of a valued ornament, it may experience the fate of the Cyclopædia of Surgery—dead from inanition—and an imperfect work may be left in the hands of the original purchasers. Such was the case with the *Dictionnaire des Etudes Médicales*, which expired at the termination of the fourth volume, leaving ourselves amongst the bereaved ones.

We wish our German *Confrères* would abandon this custom of issuing their works piecemeal; for it not unfrequently happens, as the *Abtheilungen* are sold separately, that a purchaser is unable to obtain some of them, and is thus saddled with an incomplete work, as has happened to ourselves more than once.

It is not our intention to examine the work of Mitscherlich in detail. In a former article, we spoke of the general character of the German works on Materia Medica and Therapeutics; and commended them for paying much more attention to the therapeutical relations, rather than exhausting—as is commonly the case with the English works on the subject—every topic of chemistry and natural history that has any bearing on the matter, and passing over the therapeutical portion in a very cursory and often slovenly manner. Such works are assuredly not desirable as accompaniments to a course of medical lectures. Practical materia medica cannot be completely taught by lectures, although they afford the student most important and essential facilities. An acquaintance with the sensible qualities of drugs can only be attained fully by handling them; but the principles of general therapeutics, and the indications which special articles are capable of fulfilling, can be conveyed by lectures in a manner to render the impression forcible and enduring; and the most valuable accompaniments to the student of materia medica, in the shape of books, are those that teach just so much of the sensible properties of the articles of the materia medica as may enable the practitioner to recognize and select the genuine, and to discard the spurious and imperfect; whilst they expand copiously on the adaptation of such articles to the treatment of disease. It is, doubtless, *desirable*, that the young graduate should know every thing relating to chemistry and natural history, contained in the pages of Pereira, or Royle, or Ballard and Garrod, or in the Dispensatory of the United States by Messrs. Wood and Bache; but such knowledge, however *desirable*, cannot certainly be *indispensable.* Where, consequently, it is a matter of moment, that the aspirant for medical honours should, at an early period, be sent forth in the practical exercise of his calling, the *necessaries* should be inexorably demanded, but the *luxuries* may be postponed for after attainment.

The appearance of the *Annuaire de Thérapeutique* of M. Bouchardat we have heralded more than once. This is the seventh year of its advent, and we are pleased to learn from its author, that "its success has increased year by year, so that it has now attained a circulation equalled by few works on medicine or pharmacy." "I take to myself"—he adds—"more and more credit for having borrowed of M. Arago the idea of terminating each volume by one or more unpublished works, to which I devote all my attention; and I am of opinion, that these memoirs have contributed much to cause the *Annuaire* to be sought after; so that my publisher has been obliged to reprint three years that were exhausted." p. v.

To such memoirs contained in the present number, we shall restrict our reference at present. The body of the *Annuaire*, like that of Ranking and of Braithwaite, is indeed a kind of Collectanea or Adversaria, similar to the Record department of our own Journal, and therefore does not admit of analysis. Many of the articles have indeed been already published in those *Recueils.*

Our readers are aware, that, according to the views of Liebig, aliments admit of classification into the azoted or nitrogenized, or such as are capable of forming organized tissues; and the non-organized or non-nitrogenized, such as are inservient only to respiration—views, which we have always considered not only to demand proof, but to be, in some respects, unsupported by observation; and to be based rather on chemical than physiological results. From the chemists they have received, however, great attention, and MM. Bouchardat and

Sandras in the '*Annuaires*' for 1843 and 1845, and in the supplement to that of 1846, examined into the digestion of fatty, saccharine and amylaceous substances, and endeavoured to discover the *rôle* of these substances in nutrition; and to complete the inquiry, they have, in the *Annuaire* before us, examined the "Digestion of Alcoholic Drinks;" and after a detail of certain experiments on animals and man, have made the following conclusions:—

"By comparing and associating the results of the experiments which we have just detailed, a clear idea may be formed of the mode of absorption of alcoholic drinks, the changes induced by them in the animal economy, and the *rôle* which they play in nutrition. We may begin by remarking, that for alcoholic drinks the first period of digestion, properly so called, which consists in a solution, is wanting, as it is also wanting in the digestion of fatty bodies. Alcoholic drinks undergo no other alteration in the digestive apparatus than that of being diluted by the gastric juice and mucus, the saliva, and the other fluids that may be poured into the digestive apparatus. The absorption of alcoholic drinks is effected, as Magendie had already shown, by the orifices [?] of the veins. It is especially in the stomach that this absorption takes place, when alcoholic drinks are given either in great excess or mixed with sugar. This absorption may be continued through the remainder of the intestines.

"The chyliferous vessels contribute, in no respect, to the absorption of alcoholic drinks. After they have been taken, the chyle may be very abundantly collected, if they have been given with fatty aliments: in such case the chyle exhibits no appreciable trace of alcohol.

"When alcoholic drinks are introduced into the torrent of the circulation, the alcohol is not eliminated by any of the secretory apparatuses: a small proportion only is evaporated from the lungs, and may be collected with the gases and vapours which are constantly exhaled from that organ.

"If the alcohol is introduced into the circulating apparatus in too great quantity, the arterial blood preserves the colour proper to venous blood; and the alcohol may occasion all the phenomena of asphyxia.

"The alcohol, under the influence of the oxygen incessantly introduced into the economy by respiration, may be immediately converted into water and carbonic acid. But in many of our observations we have obtained an intermedial product of combustion, *acetic acid*.

"The alcohol and the products derived from it disappear rapidly from the economy: when it is introduced simultaneously with glucose or dextrine, its destruction is more rapid than that of these last bodies." p. 280.

The results, therefore, of the observations of MM. Bouchardat and Sandras do not throw much light on the use of alcoholic agents as elements of combustion or respiration. Of late, it has been urged by Chossat and others, that when animals die of *inanitiation*, the fatal effects are owing to the fatty matters from within and without being consumed, and to the cooling influence thus induced; and it has been conceived that in long protracted fevers, alcoholic stimulants may be serviceable as calorifying agents: and that if they be properly given, they may sustain the 'vital flame' until the malignant disturbing agent or influence has passed away. It may be so: yet we think mischief is often done by over-stimulation in such cases; and the excitant has appeared to us not unfrequently to act most injuriously, by exhausting the slight amount of excitability remaining in the tissues. Life consists in a reciprocal action between special excitants and excitable membrane; and it cannot be too strongly borne in mind, that we may exhaust that excitability by the very agents employed to arouse it; and that life may really be sooner extinguished by the very means we employ to maintain it. As we sooner exhaust the feeble fire by endeavouring to fan it into greater vigour, so may we produce, by excitants, a flickering of the *flamma vitalis*, the *thermum emphytum* or *Biolychnium* prior to its more speedy extinction.

The "notice of the principal counterpoisons and the remarks on the Therapeutics of poisoning by M. Bouchardat" we pass by for the present, as we design to insert it *in toto* in the Record department of our next number.

---

*The Medical Student's Vade Mecum, or Manual of Examinations upon Anatomy, Physiology, Chemistry, Materia Medica, Surgery, Obstetrics, Practice of Medicine, Poisons, &c. Second Edition, revised and greatly enlarged.* By George Mendenhall, M. D., Lecturer on Pathology in the Medical Institute of Cincinnati, etc. 12mo. pp. 574. Lindsay and Blakiston: Philadelphia, 1847.

We spoke in suitable terms of commendation of this work on the appearance of the first edition, and are glad to find that the present is even more valuable than the former. About one hundred and fifty pages of matter has been added, embracing some subjects entirely omitted in the first edition, whilst "others have been rendered more full." For the purposes of a medical student, we regard it as decidedly the best manual of examinations in our language.

---

*The Virginia Springs, with their Analysis; and some remarks on their character, together with a Directory for the use of the White Sulphur Water, and an account of the diseases to which it is applicable, etc., and an account of the different Routes to the Springs.* By John J. Moormann, M.D., Resident physician at the White Sulphur Springs. 12mo. pp. 219. Lindsay & Blakiston: Philadelphia, 1847.

The Sulphur Springs of Virginia are among the most remarkable mineral springs of the country, and indeed of the world. Whether we regard the active constituents of the waters which render them medicinal, the mountainous region in which they are situated, and the varied and picturesque scenery along the road which the visiter from almost every quarter travels to arrive at them, we discover hygienic influences of the most powerful character, to impress anew the exhausted energies of the valetudinarian. Besides sulphur and the salts of lime, soda, magnesia, iron, &c., present in the waters of most of them, in various proportions, some contain iodine, and various gases, as sulphuretted hydrogen, carbonic acid, etc., and vary in temperature from 49°, as at the "Salt Sulphur," in Monroe, to 106°, at the "Hot Springs," in Bath County.

Dr. Moormann's work, although professedly an account of the different springs of Virginia, is mainly occupied with an account of the "White Sulphur," at which he resides, and a critical notice of a work on "The Mineral Springs of Western Virginia," by William Burke, formerly, and perhaps now, "Proprietor of the Red Sulphur Springs." In this part of the volume, Dr. M. commits the common error of all disputants, of supposing that the public take the same interest in personal controversies as the individuals themselves—otherwise, the book contains much to interest the seeker after health, who may be thinking of journeying to the mountains and springs of Virginia, and might, we think, with a little effort, and some self-denial, have been made to include much more, even without extending its pages. As it is, however, the physician will find in the analyses of the various Springs information which will enable him to judge of their adaptation to the conditions of patients laboring under derangements for which they are visited, whilst the public will learn from it the different routes and distances to be travelled to arrive at the several Springs.

---

*Water versus Hydropathy ; or an Essay on Water and its true relations to Medicine.* By EDWARD HARTSHORNE, M. D.

This is a *brochure* of 131 pages, duodecimo, in which the author has attempted to show the general therapeutic properties of cold water, and the classes of cases to which, internally and externally, it is adapted. Although the advocate for a less restricted use of this simple but energetic agent, in the cure of diseases, than the mass of physicians probably are, *at least in practice,* he very justly ridicules the exclusive reliance upon it which characterizes the modern tribe of hydropathists. The book contains many facts and useful suggestions which may not always be present to the mind of the experienced physician, whilst they cannot but be instructive to the tyro ; and to the latter, in particular, we commend its perusal, as well for the reasons we have stated, as the example of thoughtful inquiry it exhibits, on the part of one but recently from their ranks.

---

*The Diseases of Females ; including those of pregnancy and childbed.* By FLEETWOOD CHURCHILL, M. D., author of "the Theory and Practice of Midwifery," etc. etc. Fourth American edition, with illustrations. With notes by ROBERT M. HUSTON, M. D., etc. etc. 8vo. pp. 600. Lea & Blanchard, Philadelphia, 1847.

The appearance of a fourth edition of the work of Dr. Churchill on the Diseases of Females, in so brief a space of time, is high evidence of its general approval by the physicians of the United States.

One great and distinguishing trait of the volume is, that it embraces *all* the diseases peculiar to women, which can hardly be said of any other publication, and it is this circumstance that especially commends it to practitioners who are without the opportunity of consulting numerous works. In the present edition, some additions have been made by the editor, and some errors of the press have been corrected, although it is to be regretted that others have been allowed to escape.

